# Rapid Pathogen Identification in Aqueous Humor Samples by Combining Fc-MBL@Fe_3_O_4_ Enrichment and Matrix-Assisted Laser Desorption Ionization–Time of Flight Mass Spectrometry Profiling

**DOI:** 10.1128/spectrum.01767-22

**Published:** 2022-11-08

**Authors:** Jun Ren, Menghuan Yu, Wenjin Gao, Chuanfan Ding, Shengjie Li, Shaoning Yu, Wenjun Cao

**Affiliations:** a Clinical Laboratory, Eye & ENT Hospital, Fudan University, Shanghai, China; b Zhejiang Provincial Key Laboratory of Advanced Mass Spectrometry and Molecular Analysis, Institute of Mass Spectrometry, School of Material Science and Chemical Engineering, Ningbo University, Ningbo, Zhejiang, China; Georgia Institute of Technology

**Keywords:** aqueous humor, MALDI-TOF MS, magnetic enrichment, pathogen identification

## Abstract

Prompt clinical diagnosis and antimicrobial therapy are key to managing infective endophthalmitis. The small volume of aqueous humor, low bacterial counts, and empirical medication by physicians make existing diagnostic methods time-consuming and imprecise. Here, we investigated the feasibility of combining Fc-containing mannose-binding lectin-coated Fe_3_O_4_ (Fc-MBL@Fe_3_O_4_) enrichment with matrix-assisted laser desorption–ionization time of flight mass spectrometry (MALDI-TOF MS) profiling to identify pathogens in aqueous humor. Aqueous humor aspirated from freshly enucleated porcine eyes was treated with different inocula of Staphylococcus aureus, Staphylococcus epidermidis, and Klebsiella pneumoniae. We performed identification directly in aqueous humor samples and after short-term culture of micro-LB broth. Aqueous humor endophthalmitis samples were enriched with Fc-MBL@Fe_3_O_4_ and analyzed using MALDI-TOF MS. The identification time and minimum bacterial concentration required for identification were determined. The enrichment efficiency of Fc-MBL@Fe_3_O_4_ for different bacteria was greater than (87.5 ± 5.0)%. The objects of direct identification include live bacteria and bacteria treated with antibiotics, which can be completed within 1.5 h. The minimum number of bacteria needed for positive identification was 2.20 × 10^6^ CFU. For micro-LB broth culture, the identification of bacteria can be completed within 6.5 to 9.5 h for aqueous humor samples with an initial bacterial count of tens to hundreds.

**IMPORTANCE** Fc-MBL@Fe_3_O_4_ capture not only live bacteria in aqueous humor but also bacteria inactivated by antibiotics. Fc-MBL@Fe_3_O_4_ combined with micro-LB broth culture significantly reduced the turnaround time (TAT) by more than half a day by shortening the time required for bacterial identification. Our findings demonstrate that combining Fc-MBL@Fe_3_O_4_ enrichment with MALDI-TOF MS identification is a fast, sensitive, and efficient analytical method with great potential for identifying pathogens in aqueous humor samples.

## INTRODUCTION

Endophthalmitis is a rare but devastating eye infection that can lead to irreversible blindness in the infected eye within hours or days of the appearance of symptoms. More than 75% of endophthalmitis cases worldwide are caused by bacteria and can occur due to intraocular surgery, intraocular injections, trauma, continuous spread to adjacent structures, and endogenous transmission through the bloodstream (1, 2). Staphylococcus epidermidis and Staphylococcus aureus are the main pathogens that cause exogenous endophthalmitis; Klebsiella pneumoniae (especially in East Asia) and S. aureus are the main pathogens that cause endogenous endophthalmitis (2, 3). The diagnosis of specific pathogens in endophthalmitis is key to effective clinical treatment, including the administration of appropriate antibiotics.

Aqueous humor is present as ~0.3 mL of liquid and regenerates continuously, with a turnaround time of 100 min. Small samples of aqueous humor (0.1 mL) can be obtained by needle aspiration for microbial identification. Traditionally, microbial culture has been considered the gold standard for the diagnosis of endophthalmitis. However, traditional microbiological methods are suboptimal because a negative culture does not rule out a diagnosis, and 60% of aqueous aspirates are culture negative (3, 4). In addition, cultures typically require 24 h to several days for growth and observation, with limited sensitivity, which may delay patient treatment (5–7). PCR testing of intraocular fluid has the potential to rapidly identify pathogens in cases of endophthalmitis, including culture-negative cases. However, PCR detection of bacteria in aqueous humor samples currently requires complex expertise and high cost. Although some strategies have been adopted to shorten the identification process, more facile and economical methods are needed to meet practical clinical needs (8, 9).

In recent years, the development of matrix-assisted laser desorption ionization–time of flight mass spectrometry (MALDI-TOF MS) equipment has brought a simple, rapid, high-throughput, and low-cost identification technique to the routine identification of microorganisms in clinical microbiology laboratories (10, 11). MALDI-TOF MS has been directly applied to some clinical samples, including blood, urine, cerebrospinal fluid, pleural fluid, peritoneal fluid, synovial fluid, and vitreous body, but no study has specifically tested the application of MALDI-TOF-MS directly on aqueous humor samples in endophthalmitis (12–15). The identification of bacteria in aqueous humor by MALDI-TOF-MS alone has certain limitations. First, nonbacterial components of the aqueous humor can interfere with the identification of bacteria (16). Second, the physician’s empirical medication may interfere with the identification of bacteria. Bacteria inactivated by antibiotics cannot proliferate on the plate, and it is difficult to identify them by MALDI-TOF-MS alone (17). Finally, the MALDI-TOF-MS assay is not suitable for aqueous samples with low bacterial load, which may be the early stage of endophthalmitis (10, 14, 16, 17). Therefore, we need to combine special sample preparation protocols with mass spectrometry to overcome these limitations.

Functionalized magnetic nanoparticles (MNPs) have been used to capture bacteria in blood and urine due to their good biocompatibility and ease of use (18, 19). Mannose-binding lectin (MBL) is a calcium regulation-dependent carbohydrate-binding protein that can bind to carbohydrate structures on the surface of pathogenic microorganisms. Covalent coating of a genetically engineered form of the human opsonizing protein MBL linked to the flexible neck of the Fc portion of IgG1 (Fc-MBL) can clear a wide range of unspecified pathogens and endotoxins from the blood (20). Specific aptamers and antibodies conjugated to MNPs can enrich certain bacteria or some microorganisms from suspension (21–23). Bacteria in liquid media can be enriched by a novel functionalized magnetic nanoparticle (Fc-MBL@Fe_3_O_4_) and identified by MALDI-TOF MS (24). These studies indicated that Fc-MBL@Fe_3_O_4_ can effectively purify bacteria from clinical samples or liquid culture media.

To apply this technique to clinical samples with different degrees of infection, we used Fc-MBL@Fe_3_O_4_ combined with MALDI-TOF MS to establish a comprehensive and effective method for detecting bacteria in an *in vitro* model of endophthalmitis. The method includes the direct capture of live bacteria and antibiotic-inactivated bacteria from aqueous humor samples by Fc-MBL@Fe_3_O_4_ and short-term incubation of aqueous humor samples before the use of Fc-MBL@Fe_3_O_4_ to capture and identify bacteria.

## RESULTS

### Specificity of magnetic beads.

**FIG 1:**
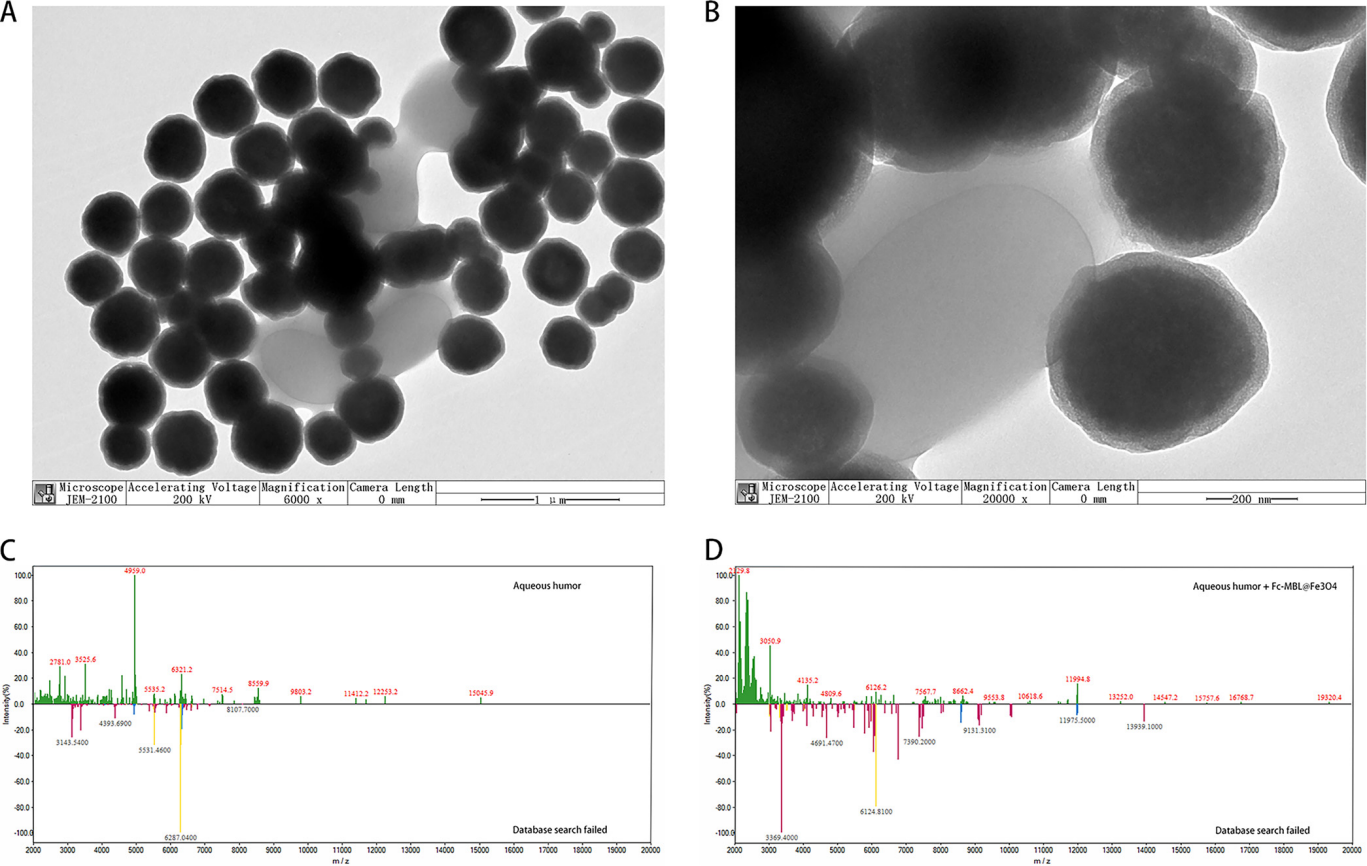
Specificity of magnetic beads. (A, B) TEM images of S. aureus conjugated with Fc-MBL@Fe_3_O_4_. (C) Mass spectrum of aqueous humor. (D) Mass spectrum of aqueous humor after enrichment with Fc-MBL@Fe_3_O_4_.

Our previous study introduced the synthetic process for Fc-MBL@Fe_3_O_4_ and the characterization of Fc-MBL@Fe_3_O_4_ (24, 25). Transmission electron microscopy (TEM) images of the binding of Fc-MBL@Fe_3_O_4_ to S. aureus are shown in Fig. 1A and B. As shown in Fig. 1C and D, characteristic peaks do not appear in the mass spectrum after Fc-MBL@Fe_3_O_4_ enrichment of aqueous humor, indicating that the components of the aqueous humor could be washed away by magnetic separation. These results indicate the feasibility of the method.

### Pathogen identification from aqueous humor by Fc-MBL@Fe_3_O_4_-binding MALDI-TOF MS.

**FIG 2:**
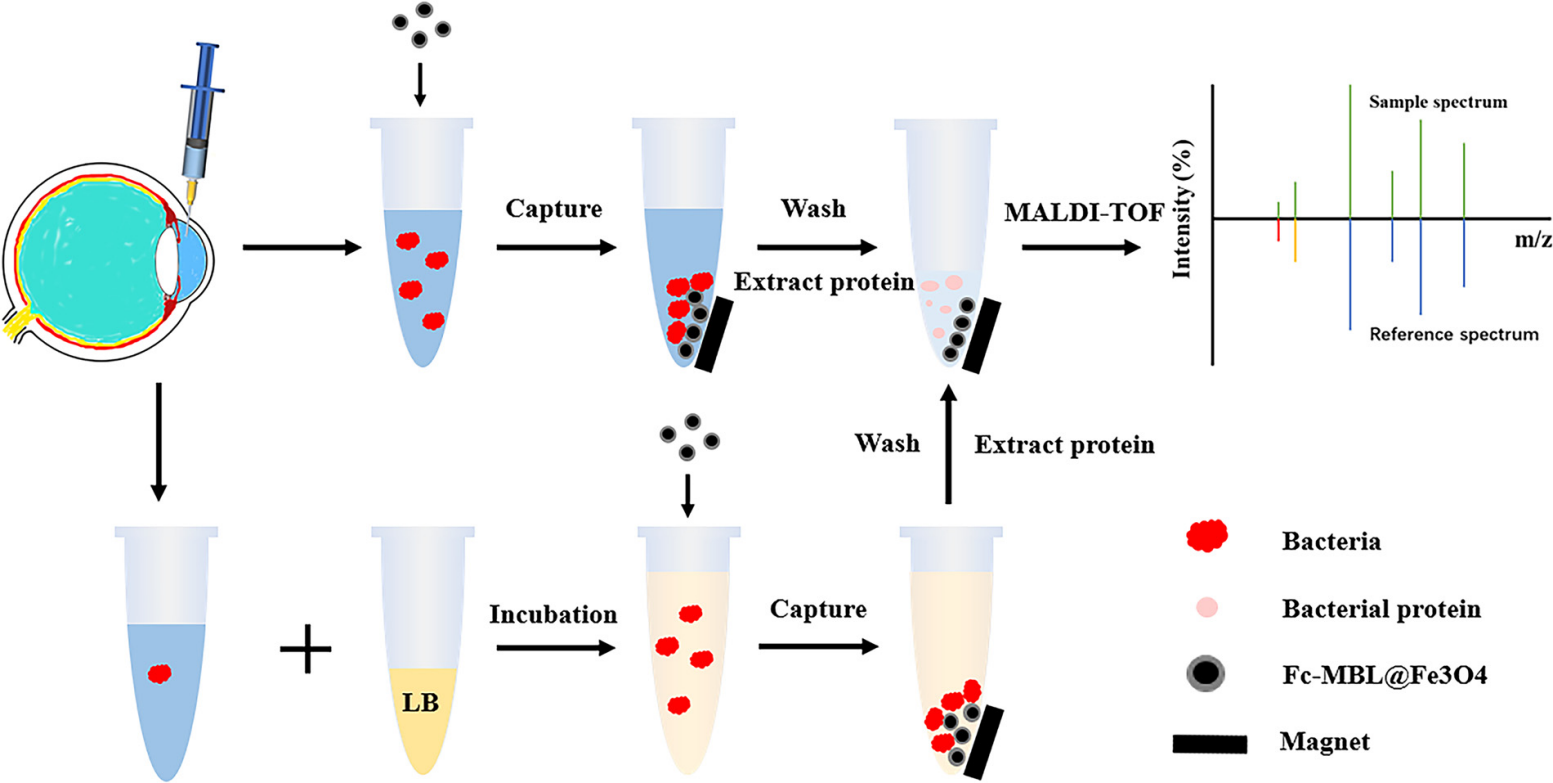
Workflow for the identification of pathogens from an aqueous humor sample.

**FIG 3:**
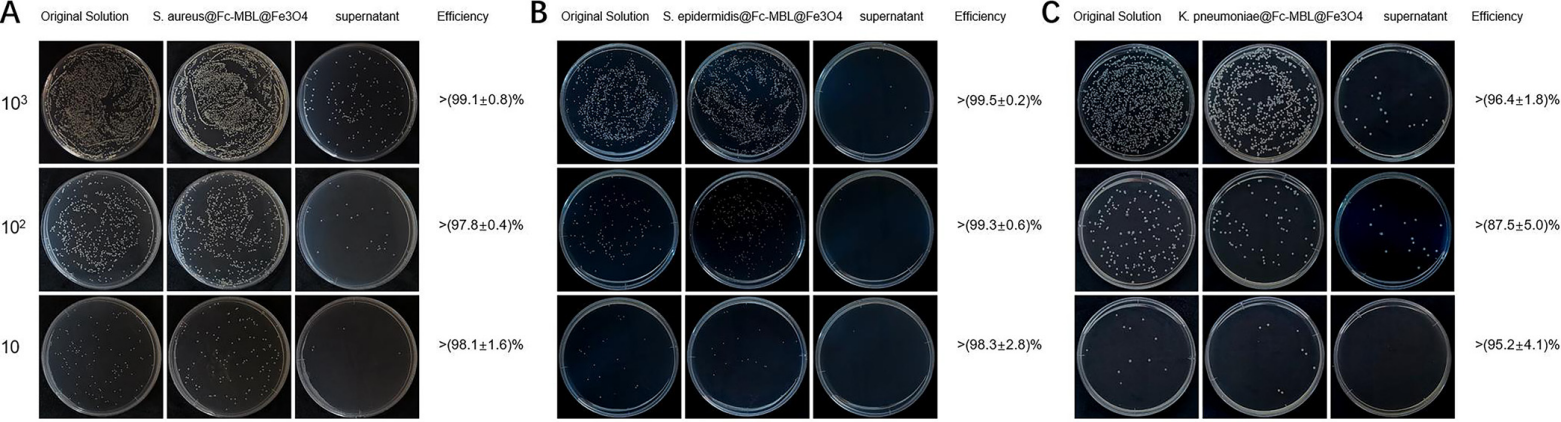
Enrichment efficiency of Fc-MBL@Fe_3_O_4_. Photographs of culture plates of the original solution, enriched pellets, and supernatant for (A) S. aureus, (B) S. epidermidis, and (C) K. pneumoniae. Bacteria were enriched from aqueous humor using Fc-MBL@Fe_3_O_4_.

The workflow of the proposed method is illustrated in Fig. 2. Before identifying the bacteria, we investigated the efficiency of magnetic beads to enrich bacteria in aqueous humor. Different concentrations of a bacterial solution (~10, ~100, and ~1,000 CFU/mL; 10 μL each) were incubated with 40 μL of aqueous humor and enriched with Fc-MBL@Fe_3_O_4_. The capture efficiency of Fc-MBL@Fe_3_O_4_ was evaluated using the plate counting method. The original bacterial solution, enriched pellet, and supernatant were cultured on a tryptic soy agar (TSA) plate for 12 to 14 h; the results are shown in Fig. 3. The enrichment efficiency of Fc-MBL@Fe_3_O_4_ for different bacteria was >(87.5 ± 5.0)% (see Table S1 in the supplemental material). The capture efficiency of the magnetic beads was higher for Gram-positive bacteria than for Gram-negative bacteria.

**FIG 4:**
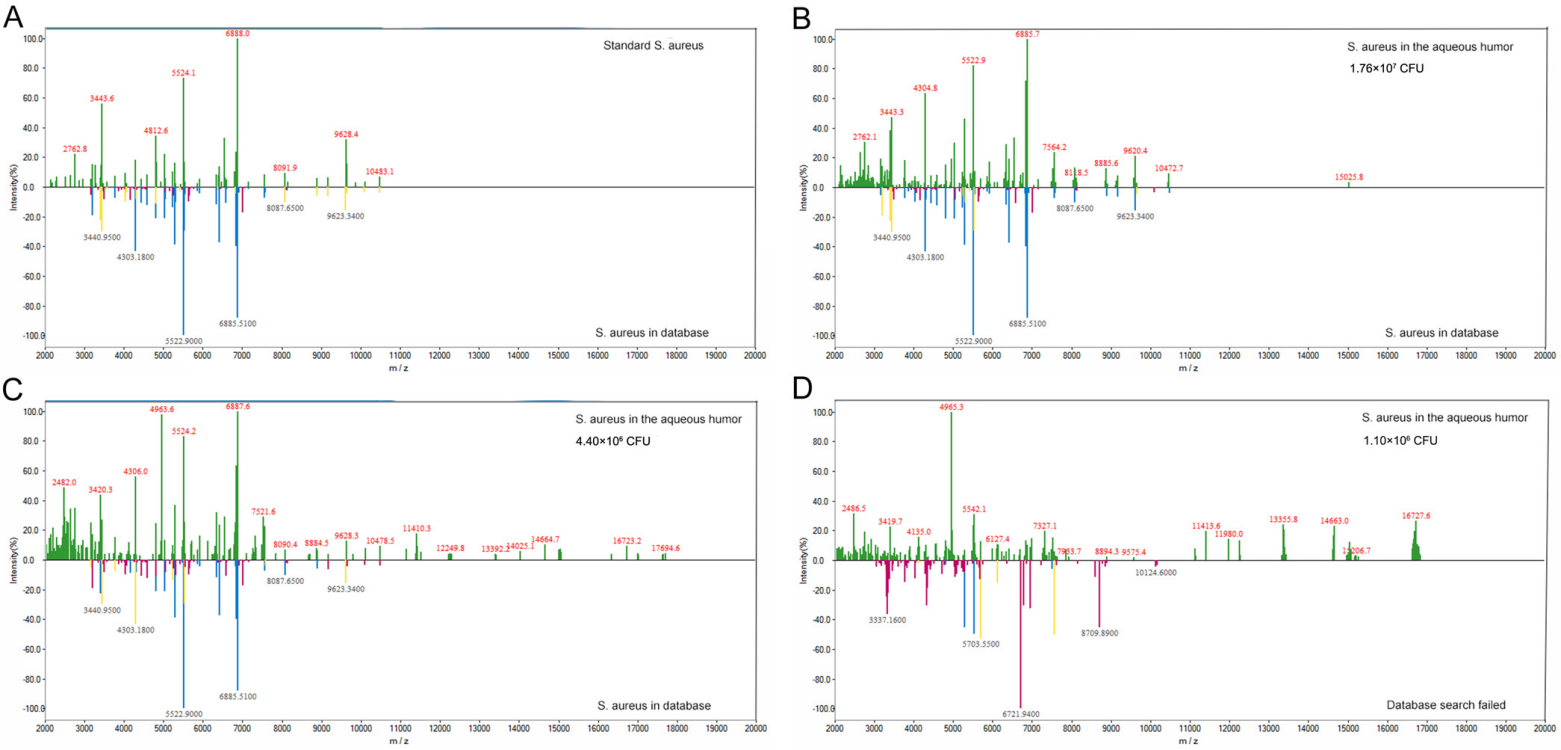
Characteristic peaks of the standard spectrum of pure S. aureus ATCC 25923 (A) versus those of different S. aureus counts: 1.76 × 10^7^ CFU (B), 4.40 × 10^6^ CFU (C), and 1.10 × 10^6^ CFU (D). The mass spectra of S. aureus were obtained from pure solution and enriched by Fc-MBL@Fe_3_O_4_ from 40 μL of aqueous humor.

**FIG 5:**
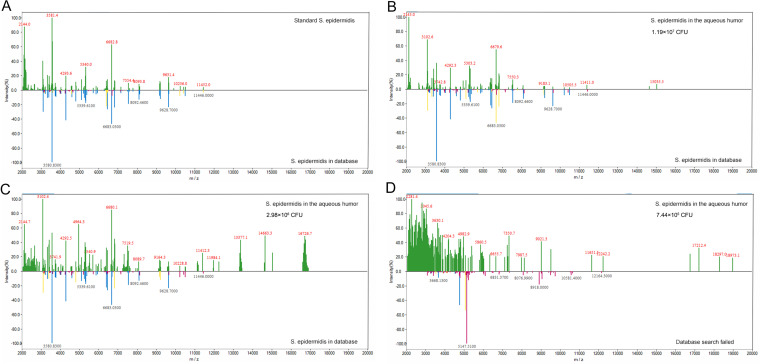
Characteristic peaks of the standard spectrum of pure S. epidermidis ATCC 12228 (A) versus those of different S. epidermidis counts: 1.19 × 10^7^ CFU (B), 2.98 × 10^6^ CFU (C), and 7.44 × 10^6^ CFU (D). The mass spectra of S. epidermidis were obtained from pure solution and enriched by Fc-MBL@Fe_3_O_4_ from 40 μL of aqueous humor.

**FIG 6:**
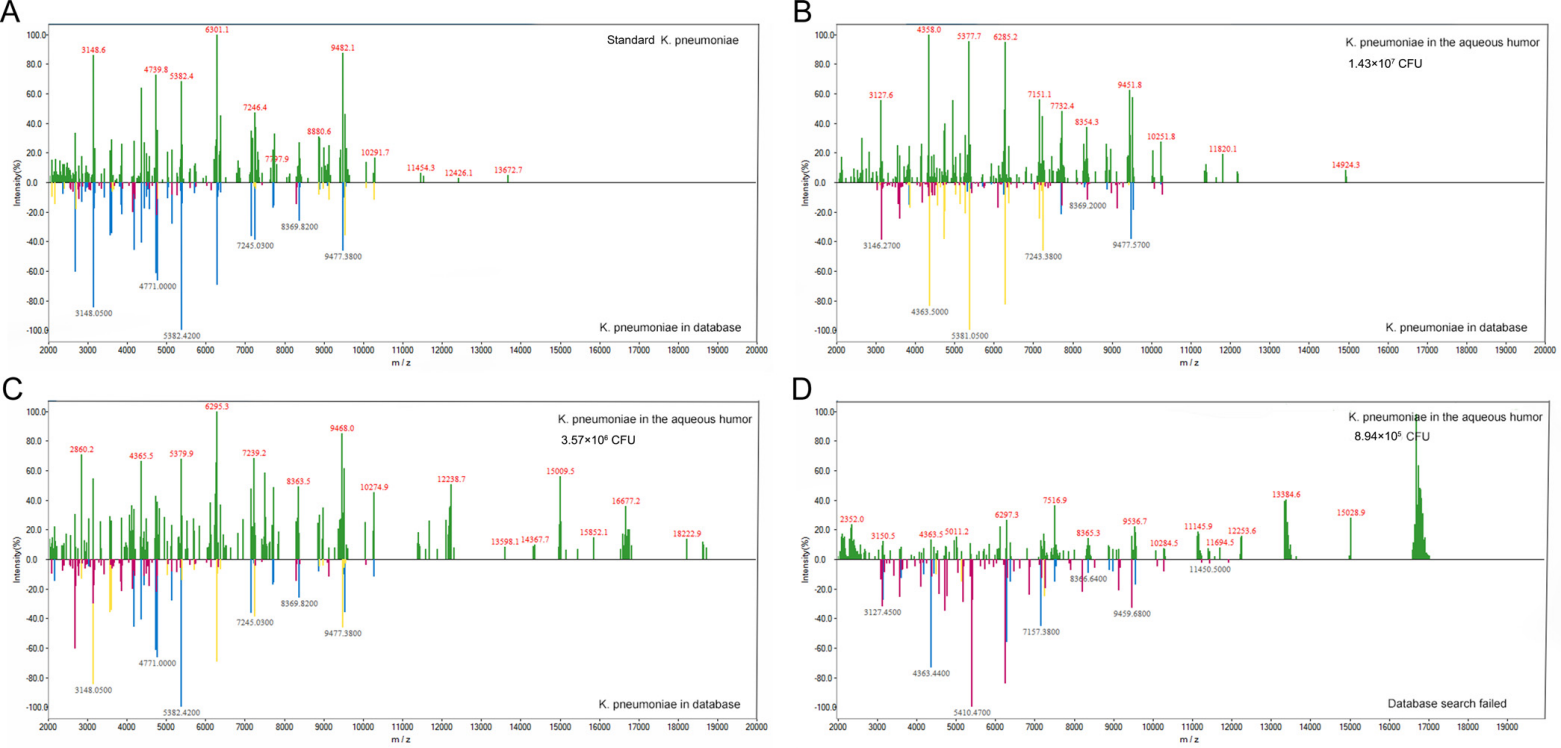
Characteristic peaks of the standard spectrum of pure K. pneumoniae CICC 21519 (A) versus those of different K. pneumoniae counts: 1.43 × 10^7^ CFU (B), 3.57 × 10^6^ CFU (C), and 8.94 × 10^6^ CFU (D). The mass spectra of K. pneumoniae were obtained from pure solution and enriched by Fc-MBL@Fe_3_O_4_ from 40 μL of aqueous humor.

To evaluate the limits of detection of the MPNs binding MALDI-TOF MS, different amounts of bacteria in 40 μL aqueous humor were enriched with Fc-MBL@Fe_3_O_4_ and identified by MALDI-TOF MS. All 15 sampling spots could be identified (100%) when the concentration of the bacterial suspension was 4.4 × 10^6^ CFU or more (Table 1). All sample identification scores are listed in Tables S2 to S4. The minimum detectable levels of S. aureus, S. epidermidis, and K. pneumoniae suspensions in aqueous humor were 2.20 × 10^6^ CFU (73.3%), 1.49 × 10^6^ CFU (40.0%), and 1.78 × 10^6^ CFU (80.0%), respectively. For centrifugal enrichment, when the bacterial suspension concentration was 8.8 × 10^6^ CFU or greater, 15 sampling points could be identified (100%). The minimum detectable levels of S. aureus, S. epidermidis, and K. pneumoniae suspensions in aqueous humor were 4.40 × 10^6^ CFU (20%), 5.95 × 10^6^ CFU (100.0%), and 3.57 × 10^6^ CFU (6.7%), respectively. The detection limit of Fc-MBL@Fe_3_O_4_ enrichment was better than that of centrifugal enrichment. For S. aureus, the detection rate was consistent in triplicate with four bacterial numbers (1.76 × 10^7^ CFU, 8.8 × 10^6^ CFU, 4.4 × 10^6^ CFU, and 1.1 × 10^6^ CFU). At 2.2 × 10^6^ CFU, there was no significant difference in the detection rate among the three replicates (*P* = 1). For S. epidermidis, the detection rate was consistent in triplicate with four bacterial numbers (1.19 × 10^7^ CFU, 5.95 × 10^6^ CFU, 2.98 × 10^6^ CFU, and 7.44 × 10^5^ CFU). At 1.49 × 10^6^ CFU, there was no significant difference in the detection rate among the three replicates (*P* = 0.8). For K. pneumoniae, the detection rate was consistent in triplicate with four bacterial numbers (1.43 × 10^7^ CFU, 7.15 × 10^6^ CFU, 3.57 × 10^6^ CFU, and 8.94 × 10^5^ CFU). At 1.78 × 10^6^ CFU, there was no significant difference in the detection rate among the three replicates (*P* = 0.286) (Table 1). The pure bacteria were tested using MALDI-TOF MS, and the obtained spectra were compared to those of bacteria captured by Fc-MBL@Fe_3_O_4_ (Fig. 4 to 6). The spectra of ≥10^7^ CFU of bacteria in aqueous humor enriched with Fc-MBL@Fe_3_O_4_ show profiles similar to the standard spectrum of pure bacteria. As the number of bacteria decreased, the intensity of the characteristic peak became smaller and smaller. The mass spectra of low numbers of S. aureus cells showed weak specific peaks at *m*/*z* = 6,885, 5,522, 4,303, and 3,440 (Fig. 4). The mass spectra of low numbers of S. epidermidis cells showed weak specific peaks at *m*/*z* = 9,628, 8,092, 6,683, 5,339, and 3,580 (Fig. 5). Finally, the mass spectra of low numbers of K. pneumoniae cells showed weak specific peaks at *m*/*z* = 9,477, 8,369, 7,245, 5,382, 4,471, and 3,148 (Fig. 6).

**TABLE 1:** Results of direct MALDI-TOF mass spectrometric analysis of bacteria in aqueous humor^*a*^

Species	No. of bacteria (CFU)^*b*^	Data for enrichment with Fc-MBL@Fe_3_O_4_					Data for centrifugation enrichment				
		No. of spots identified^*c*^			%	*P* value^*d*^	No. of spots identified			%	*P* value
S. aureus	1.76 × 10^7^	5	5	5	100		5	5	5	100	
	8.80 × 10^6^	5	5	5	100		5	5	5	100	
	4.40 × 10^6^	5	5	5	100		0	2	1	20	0.725
	2.20 × 10^6^	3	4	4	73.3	1	0	0	0	0	
	1.10 × 10^6^	0	0	0	0		0	0	0	0	
S. epidermidis	1.19 × 10^7^	5	5	5	100		5	5	5	100	
	5.95 × 10^6^	5	5	5	100		5	5	5	100	
	2.98 × 10^6^	5	5	5	100		0	0	0	0	
	1.49 × 10^6^	1	3	2	40	0.8	0	0	0	0	
	7.44 × 10^5^	0	0	0	0		0	0	0	0	
K. pneumoniae	1.43 × 10^7^	5	5	5	100		5	5	5	100	
	7.15 × 10^6^	5	5	5	100		5	5	5	100	
	3.57 × 10^6^	5	5	5	100		1	0	0	6.7	1
	1.78 × 10^6^	5	5	3	86.7	0.286	0	0	0	0	
	8.94 × 10^5^	0	0	0	0		0	0	0	0	

a Each sample was analyzed three times, each time adding 5 parallel spots on the target plate.

b The standard deviations for the S. aureus, S. epidermidis, and K. pneumoniae CFU were 10.8, 5.3, and 12.5 CFU, respectively.

c Out of 5 target spots.

d Where no *P* value is given, no statistics were computed because the detection rate in triplicate was a constant. Based on the data type and distribution, Fisher’s exact test was used to compare groups.

The bacteria treated with antibiotics detected by Fc-MBL@Fe_3_O_4_ combined with MALDI-TOF MS were studied. The original bacterial solution and bacteria treated with different concentrations of antibiotics were cultured on a Mueller-Hinton (MH) agar plate for 24 h, and photographs were taken (Fig. S1). Antibiotic concentrations were referenced to the bacterial MIC fold point in the CLSI manual and demonstrated that >99.9% of bacteria were killed by antibiotics according to the plate counts (26). Scanning electron microscopy (80,000×) showed that S. aureus cells were spherical after 24 h of vancomycin (64 μg/mL) treatment, with an intact cell but depressions on the surface (Fig. S2). The complete bacterial structure showed that inactivated bacteria can be enriched by Fc-MBL@Fe_3_O_4_ and identified using MALDI-TOF MS. Fc-MBL@Fe_3_O_4_ enriched bacteria in aqueous humor containing antibiotics, with successful identification by MALDI-TOF MS after treatment with 10 μL of acetonitrile (ACN)-formic acid (50/50) solution. Detailed antibiotic concentrations and identification results are provided in Table 2. All sample identification scores are listed in Table S5. The percent of S. aureus cells (4.96 × 10^6^ CFU) and S. epidermidis cells (3.16 × 10^6^ CFU) treated with vancomycin and erythromycin that were identified was >60%. The percent of K. pneumoniae cells (7.71 × 10^6^ CFU) treated with tobramycin that were identified was 100%. Fc-MBL@Fe_3_O_4_ combined with mass spectrometry can still effectively complete bacterial identification for low bacterial count samples treated with antibiotics.

**TABLE 2:** Results of MALDI-TOF MS analysis of bacteria in aqueous humor after antibiotic action^*a*^

Species	Data for:								
	Erythromycin			Vancomycin			Tobramycin		
	Concn (μg/mL)	No. of spots identified^*b*^	%	Concn (μg/mL)	No. of spots identified	%	Concn (μg/mL)	No. of spots identified	%
Staphylococcus aureus	16	3	60	8	5	100			
	32	3	60	16	4	80			
	64	5	100	32	5	100			
Staphylococcus epidermidis	8	4	80	8	3	60			
	16	2	40	16	5	100			
	32	4	80	32	5	100			
Klebsiella pneumoniae							7	5	100
							14	5	100
							28	5	100

a Five parallel MALDI target spots were analyzed.

b Out of 5 target spots.

The time required for sample processing was 50 min. The dripping and drying time of the sample and CHCA (α-cyano-4-hydroxycinnamic acid) on the target plate was 30 min. The time required to analyze a single spot on the target plate by MALDI-TOF MS was less than 60 s. Overall, the time for pathogen identification with MALDI-TOF MS, from sample processing to the acquisition of results, was less than 1.5 h for a single sample.

### Micro-LB broth for short-term expansion of bacteria in aqueous humor.

Micro-LB broth significantly amplified the number of bacteria within a few hours, based on the counts of the plate colonies before and after bacterial incubation (Table 3). The initial S. aureus quantity was 10 to 100 CFU, which was expanded to 10^5^ CFU after 5 h of culture in different volumes (10, 20, 30 μL) of LB broth and to 10^6^ CFU after 6 h. The initial K. pneumoniae quantity was 10 to 100 CFU, which was expanded to 10^4^ CFU after 5 h of culture in different volumes (10, 20, 30 μL) of LB broth and to 10^5^ CFU after 6 h. The initial S. epidermidis quantity was 10 to 100 CFU, which was expanded to 10^5^ CFU after 7 h of culture in different volumes (10, 20, 30 μL) of LB broth and to 10^6^ CFU after 8 h. We performed two replicate experiments, using the percentage identified as the evaluation criterion. All sample identification scores are listed in Table S6. S. aureus cells were cultured in three different volumes of LB broth for 6 h, all of which were enriched with magnetic beads and identified using mass spectrometry, but the percentage of identification obtained using 20 μL LB broth (100%) was better than that obtained with 10 μL (40%) or 30 μL LB broth (46.7%) when cultured for 5 h. For S. aureus, 20 μL LB broth is more suitable. When K. pneumoniae cells were cultured for 5 h, the identification percentages obtained using 10 μL (20%) and 20 μL (20%) LB broth were better than that obtained using 30 μL LB broth (6.7%). When K. pneumoniae cells were cultured for 6 h, the identification percentages obtained using 20 μL (100%) and 30 μL (100%) LB broth were better than that obtained with 10 μL LB broth (93.3%). For K. pneumoniae, 20 μL LB broth is more suitable, because the lowest percentage of identification was obtained when K. pneumoniae cells were cultured in 10 μL LB broth for 6 h. When S. epidermidis cells were cultured for 7 h, the percentages of identification obtained using 20 μL (26.7%) and 30 μL (20%) LB broth were better than that obtained with 10 μL LB broth (0%). When S. epidermidis cells were cultured for 8 h, the percentages of identification obtained using 30 μL (100%) and 20 μL (93.3%) LB broth were better than that obtained with 10 μL LB broth (53.3%). The smaller the volume of culture medium, the better the detection of the optical density (OD) value; thus, 20 μL LB broth is more suitable for the cultivation of S. epidermidis cells.

**TABLE 3:** Results of MALDI-TOF MS analysis of micro-LB broth for short-term incubation of bacteria^*a*^

Species (amt of LB [μL])	Results by incubation time									
	5 h					6 h				
	Preincubation (CFU)	Postincubation (CFU)	No. of spots identified^*b*^	%	*P* value^*c*^	Preincubation (CFU)	Postincubation (CFU)	No. of spots identified	%	*P* value
S. aureus (10)	49 ± 2	(6.70 ± 0.56) × 10^4^	0	40	0.126	49 ± 2	(1.48 ± 0.06) × 10^6^	5	100	
	80 ± 5	(3.63 ± 0.08) × 10^5^	3			80 ± 5	(2.79 ± 0.10) × 10^6^	5		
	52 ± 7	(1.43 ± 0.12) × 10^5^	3			52 ± 7	(1.34 ± 0.12) × 10^6^	5		
S. aureus (20)	49 ± 2	(2.97 ± 0.13) × 10^5^	5	100		49 ± 2	(3.42 ± 0.14) × 10^6^	5	100	
	80 ± 5	(4.93 ± 0.38) × 10^5^	5			80 ± 5	(4.63 ± 0.59) × 10^6^	5		
	52 ± 7	(3.03 ± 0.07) × 10^5^	5			52 ± 7	(2.39 ± 0.17) × 10^6^	5		
S. aureus (30)	49 ± 2	(3.98 ± 0.23) × 10^5^	0	46.7	0.009	49 ± 2	(4.14 ± 0.22) × 10^6^	5	100	
	80 ± 5	(6.28 ± 0.34) × 10^5^	5			80 ± 5	(3.28 ± 0.30) × 10^6^	5		
	52 ± 7	(5.68 ± 0.24) × 10^5^	2			52 ± 7	(4.60 ± 0.16) × 10^6^	5		
K. pneumoniae (10)	21 ± 1	(8.40 ± 0.70) × 10^3^	0	20	0.066	21 ± 1	(2.04 ± 0.10) × 10^5^	4	93.3	1
	34 ± 2	(1.76 ± 0.06) × 10^4^	3			34 ± 2	(1.97 ± 0.12) × 10^5^	5		
	39 ± 4	(1.87 ± 0.10) × 10^4^	0			39 ± 4	(2.12 ± 0.14) × 10^5^	5		
K. pneumoniae (20)	21 ± 1	(1.64 ± 0.13) × 10^4^	2	20	0.725	21 ± 1	(2.32 ± 0.16) × 10^5^	5	100	
	34 ± 2	(1.95 ± 0.07) × 10^4^	0			34 ± 2	(2.37 ± 0.14) × 10^5^	5		
	39 ± 4	(2.02 ± 0.07) × 10^4^	1			39 ± 4	(2.56 ± 0.21) × 10^5^	5		
K. pneumoniae (30)	21 ± 1	(1.88 ± 0.11) × 10^4^	1	6.7	1	21 ± 1	(3.24 ± 0.18) × 10^5^	5	100	
	34 ± 2	(2.77 ± 0.14) × 10^4^	0			34 ± 2	(4.72 ± 0.35) × 10^5^	5		
	39 ± 4	(2.59 ± 0.16) × 10^4^	0			39 ± 4	(4.56 ± 0.27) × 10^5^	5		
	7 h					8 h				
S. epidermidis (10)	171 ± 14	(3.70 ± 0.38) × 10^4^	0	0		171 ± 14	(9.80 ± 0.87) × 10^5^	2	53.3	1
	216 ± 13	(1.70 ± 0.12) × 10^5^	0			216 ± 13	(1.02 ± 0.08) × 10^6^	3		
	282 ± 15	(1.89 ± 0.13) × 10^5^	0			282 ± 15	(1.21 ± 0.11) × 10^6^	3		
S. epidermidis (20)	171 ± 14	(2.64 ± 0.09) × 10^5^	3	26.7	0.231	171 ± 14	(1.92 ± 0.07) × 10^6^	4	93.3	1
	216 ± 13	(2.90 ± 0.09) × 10^5^	0			216 ± 13	(2.72 ± 0.08) × 10^6^	5		
	282 ± 15	(3.11 ± 0.19) × 10^5^	1			282 ± 15	(2.53 ± 0.17) × 10^6^	5		
S. epidermidis (30)	171 ± 14	(4.42 ± 0.26) × 10^5^	0	20	0.725	171 ± 14	(3.52 ± 0.23) × 10^6^	5	100	
	216 ± 13	(3.70 ± 0.21) × 10^5^	2			216 ± 13	(4.04 ± 0.17) × 10^6^	5		
	282 ± 15	(3.82 ± 0.18) × 10^5^	1			282 ± 15	(3.68 ± 0.27) × 10^6^	5		

a Five parallel MALDI target spots were analyzed. Micro-LB, LB (10 μL, 20 μL, 30 μL) + 10 μL bacterial solution + 10 μL aqueous humor.

b Out of 5 target spots.

c Where no *P* value is given, no statistics were computed because the detection rate in triplicate was a constant. Based on the data type and distribution, Fisher’s exact test was used to compare groups.

**FIG 7:**
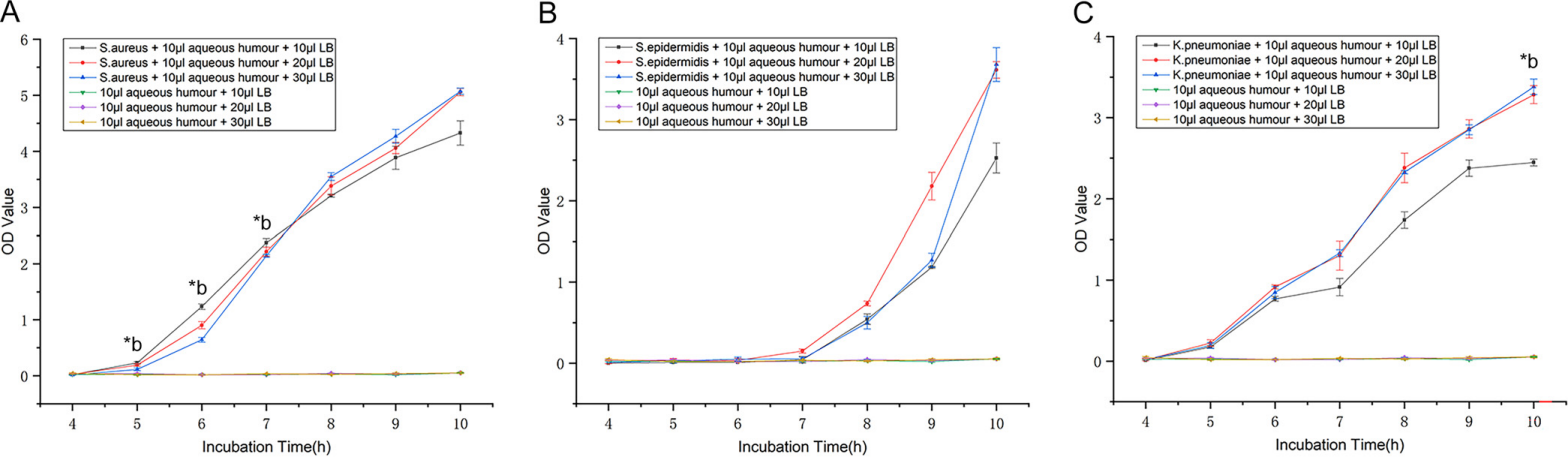
Micro-LB broth for short-term expansion of bacteria in aqueous humor. OD values of solutions containing different volumes of LB broth from 4 to 10 h: S. aureus (A), S. epidermidis (B), and K. pneumoniae (C). b, (bacteria + 10 μL aqueous humor + 10 μL LB) versus (bacteria + 10 μL aqueous humor + 30 μL LB). *, *P* < 0.05; **, *P* < 0.001.

The three bacteria were cultured in different volumes of LB broth for 10 h, and the OD at 600 nm (OD_600_) of the culture solution was measured every hour starting at 4 h. The OD_600_ values of S. aureus and K. pneumoniae started to change at 5 h, and there were significant changes at 6 h (Fig. 7A and C). The OD_600_ value of S. epidermidis began to change at 7 h, and there was a significant change at 8 h (Fig. 7B). Detailed OD values are listed in Table S7. Because the time at which the OD value changed coincided with the time when the bacteria were successfully identified, the OD value is considered useful as a reference for the short-term incubation time of bacteria. The OD values of S. aureus cells cultured in 10 μL and 30 μL LB broth for 5 h (*P* = 0.028), 6 h (*P* = 0.022), and 7 h (*P* = 0.036) were significantly different (Table S7). There was no significant difference in the OD values of S. epidermidis cells cultured in three different volumes of LB broth (*P* > 0.05). The OD values of K. pneumoniae cells cultured in 10 μL and 30 μL LB broth for 10 h (*P* = 0.034) were significantly different. Therefore, for detection of the OD value, 10-μL and 20-μL LB broth cultures are better than 30-μL broth cultures.

When the number of bacteria is tens to hundreds, the short-term bacterial culture time is 5 to 8 h, and the sample pretreatment and identification steps take about 1.5 h. Overall, using MALDI-TOF MS, pathogen identification was performed on a single sample in less than 9.5 h, from bacterial amplification in LB broth to the acquisition of results.

## DISCUSSION

Endophthalmitis is a rare but fatal eye infection that can lead to permanent loss of useful vision in the affected eye. Traditionally, to identify the causative bacteria of endophthalmitis, we would send aqueous humor samples for Gram staining and microbial culture (3, 6, 27). However, pigment granules often appear purple on Gram stain and can be mistaken for Gram-positive cocci, interfering with the rapid diagnosis of pathogens (3). Furthermore, traditional microbial culture is time-consuming, and the identification rate for pathogenic bacteria is relatively low, which may lead to delayed treatment and poor prognosis. It is challenging to design a fast, low-cost method for accurate detection of pathogenic bacteria in small-volume samples. In this study, we developed a rapid and accurate diagnostic system that combines Fc-MBL@Fe_3_O_4_ with MALDI-TOF MS to identify pathogenic bacteria in aqueous humor samples.

Aqueous humor is formed from plasma and has higher concentrations of ascorbic acid and lactate but lower concentrations of protein. It mainly contains water, carbohydrates, organic ions, amino acids, and glucose (28–30). When samples of aqueous humor from patients with endophthalmitis are taken for examination, the gel-like aqueous humor and its constituents may interfere with the MS identification of bacteria (16). A previous study showed that Fc-MBL@Fe_3_O_4_ could enrich clinically unknown bacteria from blood culture bottles for MALDI-TOF MS database identification (31). Here, we demonstrated that the enrichment efficiency of magnetic beads in aqueous humor was >(87.5 ± 5.0)%. Comparing the mass spectra of aqueous humor and aqueous humor treated with magnetic beads, we found that the main components of aqueous humor are not enriched by Fc-MBL@Fe_3_O_4_ and can be washed away by magnetic separation. Currently, there is no relevant literature report on the identification of bacteria in aqueous humor using MALDI-TOF MS. Previous studies have described an association between the bacterial concentration in the vitreous and the recognition capability of MALDI-TOF-MS. The minimum bacterial concentration required for MALDI-TOF MS identification in the vitreous is >7.889 × 10^6^ CFU/mL (17). The volume of a vitreous sample is approximately 0.5 mL. To assess the bacterial counts in vitreous samples, we replaced “CFU/μL” with “CFU/mL.” Here, we present a magnetic bead-bound MALDI-TOF-MS method that can directly identify S. aureus, S. epidermidis, and K. pneumoniae in aqueous humor without bacterial subculture. Our experiments demonstrate that the detection limit of bacteria in aqueous humor samples is approximately 10^6^ CFU. The method can be completed within 1.5 h of obtaining the aqueous humor sample from the patient. When the number of bacteria in aqueous humor samples is above the detection limit, the use of magnetic beads for direct bacterial enrichment combined with mass spectrometric identification is a rapid and effective detection method.

In up to 60% of cases, cultures of aqueous humor samples are negative (3), which may be related to the patient’s systemic and ocular medications. The use of antibiotics at patient visits may also hinder the identification of microorganisms using traditional microbiological methods (17, 32). We demonstrate that magnetic beads can enrich antibiotic-treated S. aureus, S. epidermidis, and K. pneumoniae in aqueous humor. By comparing the mass spectra of standard bacteria (strains ATCC 12228, ATCC 25923, CICC 21519) and bacteria inactivated by antibiotics, we found that the intensity of some characteristic peaks of bacteria inactivated by antibiotics is weakened. The characteristic peaks of these strains were obtained by searching public databases using software and can be seen below the mass spectra in Fig. S3 in the supplemental material. The mass spectra of dead S. aureus cells showed weak specific peaks at *m/z* = 6,885 and 5,522 (Fig. S3). The mass spectra of dead S. epidermidis cells showed weak specific peaks at *m/z* = 5,340, 6,683, 8,090, and 9,626 (Fig. S4). However, the mass spectra of dead K. pneumoniae cells showed weak specific peaks at *m/z* = 3,148, 5,382, and 6,301 (Fig. S5). There are fewer characteristic peaks in the mass spectra of dead bacteria than live bacteria. Compared to the identification of bacteria using mass spectrometry alone, this method is more suitable for clinical sample detection and can improve the positivity rate of aqueous humor samples.

A previous study showed that MALDI-TOF-MS will not work in the early stages of endophthalmitis (16, 33). Short-term bacterial culture is essential for aqueous humor samples with low bacterial loads. Traditional culture and blood culture bottle culture are often used in clinical laboratories. A previous study showed that the mean detection times for routine microbial culture and blood culture combined with MALDI-TOF MS for suspected endophthalmitis are 5.39 ± 0.56 days and 3.17 ± 0.40 days, respectively (14). To shorten the incubation time, we used micro-LB broth for incubation. We demonstrated that micro-LB broth can satisfy the short-term expansion of bacteria, and monitoring the OD_600_ value can determine the incubation time. In our study, the identification of bacteria can be completed within 6.5 to 9.5 h for aqueous humor samples with an initial bacterial count of tens to hundreds.

For aqueous humor samples in endophthalmitis, we have established a complete identification protocol, including for live bacteria and bacteria treated with antibiotics, as well as low bacterial load and high bacterial load samples. By applying this immunoaffinity MALDI-TOF MS method, pathogen analysis of aqueous humor samples can provide great specificity and high sensitivity. Further studies are needed to validate the potential clinical applications.

In our study, Fc-MBL@Fe_3_O_4_ coupled with MALDI-TOF MS could only identify bacteria at the species level and could not judge drug resistance. For drug-resistant bacteria, it is necessary to guide clinicians to use antibiotics through drug susceptibility testing. When the number of dead bacteria is below the detection limit of this method, Fc-MBL@Fe_3_O_4_-coupled MALDI-TOF MS identification will not work, because we cannot expand the number of dead bacteria by short-term culture. Currently, we use swine aqueous humor plus bacteria for simulation experiments. Our goal is to apply this method to clinical aqueous humor sample identification. Our results should be used in larger investigations of more surgical samples from endophthalmitis to confirm the findings. Because the inside of the eye is a sterile environment, the bacterial counts in most aqueous humor samples tend to be low. Compared with the direct enrichment of bacteria by magnetic beads, we believe that the enrichment of bacteria after short-term culture in micro-LB broth is more suitable for clinical aqueous humor samples.

## MATERIALS AND METHODS

### Materials.

α-Cyano-4-hydroxycinnamic acid (CHCA), acetonitrile, and formic acid were purchased from Merck (Darmstadt, Germany). Trifluoroacetic acid (TFA) was purchased from Adamas Reagent Co. Ltd. (Shanghai, China). Tris(hydroxymethyl)methyl aminomethane (Tris) was purchased from Beijing HWRK Chem Co., Ltd. (Beijing, China). Tryptic soy agar (TSA) and Luria-Bertani (LB) broth were purchased from Beijing Land Bridge Technology Co., Ltd. (Beijing, China). Sterile water was obtained by sterilization at 120°C. Calcium chloride was obtained from Shanghai Chemical Corp. (Shanghai, China). Vancomycin and erythromycin were purchased from Shanghai Macklin Biochemical Technology Co., Ltd. (Shanghai, China). Tobramycin was purchased from Aladdin Reagent Co., Ltd. (Shanghai, China). S. epidermidis (ATCC 12228), S. aureus (ATCC 25923), *K. pneumonia* (CICC 21519), and Escherichia coli (ATCC 8739) were purchased from the China Center of Industrial Culture Collection (CICC; Beijing, China) or the American Type Culture Collection (ATCC; Manassas, VA, USA). The M-Discover 100 Excellence MALDI-TOF MS was obtained from Meihua Medical Technology Limited (Zhuhai, China). The Milli-Q deionized water system was obtained from Jingyuan Science & Technology Co., Ltd. (Hangzhou, China). The spectrophotometer and bacterial incubator were obtained from Thermo Fisher Scientific (USA). The centrifuge was obtained from Eppendorf (Germany).

### Aqueous humor preparation.

Fresh porcine eyeballs were obtained from the butcher within 8 h of slaughter. First, the porcine eyes were disinfected by soaking in 5% betadine solution for 10 s; then, they were washed with sterile water and dried. A 1-mL syringe was used to extract the aqueous humor through paracentesis of the anterior chamber, and we typically obtained a volume of 100 μL per eye. The aqueous humor was filtered through a 0.22-μm filter membrane and added to a 1.5-mL centrifuge tube. The aqueous humor was irradiated with UV for 15 min and stored at −80°C until use.

### Bacterial stock preparation.

All bacterial strains were streaked onto tryptic soy agar (TSA), and the agar plates were incubated for 12 to 14 h. Single colonies were selected from the incubated plates and suspended in sterile water. The absorbance at 600 nm was measured by UV-visible (UV-vis) absorption spectrometry, and the optical density at 600 nm (OD_600_) of a single colony was adjusted to 0.7 to 0.8 to produce bacterial stock (BS). Accurate bacterial concentrations were determined by the plate counting method.

### Enrichment efficiency of Fc-MBL@Fe_3_O_4_ in aqueous humor.

BS was added to aqueous humor to mimic clinically infected samples. First, the bacterial solution was diluted to 10^5^ CFU/mL with sterile water, and 10 μL of the bacterial solution was added to 10 μL of aqueous humor with mixing. Next, 2.5 μL Fc-MBL@Fe_3_O_4_ solution (10 mg/mL) and 27.5 μL Tris-HCl solution (0.1 mM, pH 7.4) were added to 10 μL bacterial solution (10^5^ CFU/mL) and incubated at 37°C for 30 min with consistent shaking at 1,200 rpm. After magnetic separation, the supernatant was absorbed by the pipette and washed three times with 30 μL of sterile water. A 10-μL aliquot of the bacterial solution (10^5^ CFU/mL) was added to 40 μL of sterile water as the original bacterial solution. The supernatant obtained after magnetic separation and the sterile water after cleaning were collected as supernatant. The original bacterial solution, enriched Fc-MBL@Fe_3_O_4_, and supernatant were cultured on a TSA plate for 12 to 14 h. The enrichment efficiency of Fc-MBL@Fe_3_O_4_ was calculated by plate counting. The experiment was repeated by taking 10 μL bacterial solution from 10^4^ CFU/mL and 10^3^ CFU/mL, according to the above steps. Finally, the enrichment efficiency of Fc-MBL@Fe_3_O_4_ at three different bacterial concentrations was obtained. Triplicate repeats were performed in parallel for each enrichment.

### Detection limit of Fc-MBL@Fe_3_O_4_ binding MALDI-TOF MS.

After BS was diluted 10^5^ times with sterile water, 100 μL of the bacterial solution was plated onto TSA plates and incubated at 37°C for 12 to 14 h. Colony counting was performed to estimate the bacterial concentration of the original bacterial solution. BS (80 μL) was mixed into 80 μL aqueous humor to make serial dilution samples of 2 to 2^4^ (doubling dilution with aqueous humor). Fc-MBL@Fe_3_O_4_ (5 μL) and Tris-HCl solution (100 μL) was added to each diluted sample (80 μL); the samples were then incubated at 37°C for 30 min with consistent shaking at 1,200 rpm. The Fc-MBL@Fe_3_O_4_ enrichments were washed three times with 30 μL of water. The supernatant was discarded, and the Fc-MBL@Fe_3_O_4_ enrichment was air dried and resuspended in 5 μL of 70% formic acid and 5 μL of ACN. The extracted bacterial protein solution was subjected to MALDI-TOF MS analysis. The remaining diluted samples (80 μL) were centrifuged at 6,000 × *g* at room temperature for 10 min to collect bacterial cells in pellets. The supernatant was carefully discarded without disturbing the pellets. The pellet was washed three times with 100 μL sterile distilled water, resuspended in 5 μL sterile distilled water (vortexed thoroughly), and analyzed by MALDI-TOF MS. Each Fc-MBL@Fe_3_O_4_ enrichment and centrifugation enrichment was repeated three times in parallel.

### Detection of bacteria treated with antibiotics by Fc-MBL@Fe_3_O_4_ combined with MALDI-TOF MS.

Based on the clinical use of antibiotics for bacterial endophthalmitis, we selected vancomycin, erythromycin, and tobramycin. Vancomycin and roxithromycin were used against S. aureus and S. epidermidis. Tobramycin was used against K. pneumoniae. Antibiotic concentrations were referenced to the bacterial MIC fold point in the CLSI manual (26). We added 20 μL BS and 40 μL aqueous humor to 200 μL of antibiotics at different concentrations and incubated them overnight at 37°C. Accurate bacterial numbers of the BS were determined by plate counting. The incubated bacterial solution (100 μL) was plated onto a TSA board and incubated at 37°C for 12 to 14 h. A plate colony count was used to determine whether the bacteria were killed. Tris-HCl solution (100 μL) and Fc-MBL@Fe_3_O_4_ (5 μL) were added to the remaining 100 μL of the postincubation bacterial solution, and the mixture was incubated at 37°C for 30 min with shaking. The bacterial proteins were extracted with 5 μL of 70% formic acid and 5 μL ACN and analyzed using MALDI-TOF MS.

### Trace amounts of LB broth-cultured bacteria in aqueous humor.

BS was diluted 10^5^ times with sterile water; then, 10 μL bacterial solution and 10 μL aqueous humor were added to three different volumes (10 μL, 20 μL, 30 μL) of LB medium. The mixture was incubated at 37°C with shaking at 850 rpm, and the OD_600_ was measured every hour (0 to 8 h). Taking the bacterial culture time as the abscissa and the OD_600_ as the ordinate, the standard curve of the linear relationship between the bacterial culture time and the OD_600_ value was established. For bacterial identification, the culture was terminated when the OD_600_ value of the mixture changed. Each mixture was diluted with sterile water to 200 μL, and 20 μL was taken for colony counting after dilution. The remaining samples were enriched with Fc-MBL@Fe_3_O_4_ and identified by mass spectrometry. Accurate bacteria concentrations were determined by plate counting. For trace amounts of LB broth-cultured bacteria in aqueous humor, we performed three replicates.

### MALDI-TOF MS identification.

A 1.2-μL aliquot of the bacterial protein solution extracted from Fc-MBL@Fe_3_O_4_ was deposited onto a MALDI target plate calibrated with bacterial test standard ATCC 8739. Each sample was deposited on at least five parallel spots on one target. Each deposit was coated with a CHCA matrix (1 μL, 10 mg/mL in ACN/H_2_O [vol/vol = 1/1] containing 2% TFA) on the target plate and air-dried at room temperature. MALDI-TOF mass spectrometry was performed in linear positive mode. The target plates were then subjected to mass spectrometry on an M-Discover 100 Excellence mass spectrometer (Zhuhai, China) with a mass range from 2,000 to 20,000 Da. The raw mass spectra were processed using MicroCtrl version 1.0 software. The data processing parameters included spectral smoothing, baseline correction, and automatic peak finding. According to the manufacturer, a score of >2.0 is considered reliable at the species level, a score of 1.7 to 2.0 indicates identification at the genus level, and a score of <1.7 indicates an unreliable result. The scoring method of the MicroDiscovery software we used is the same as that used in Bruker Biotyper software. Each sample was deposited on at least five parallel spots on one target, and all results were randomly selected from the obtained mass spectra.

### Statistical analysis.

SPSS software version 26 (SPSS Inc., Chicago, IL, USA) was used for statistical analysis. Depending on the data type, the mean ± standard deviation (SD) or percentage were used to describe the results. Based on the data type and distribution, the Fisher’s exact test, Kruskal-Wallis test, and Bonferroni correction were used to compare groups.

### Data availability.

The MALDI-TOF MS data generated in this study have been deposited in the Zenodo database (https://zenodo.org/record/7112446#.YzFBA0xBziA).
