# Supplementary material for: Rapid Pathogen Identification in Aqueous Humor Samples by Combining Fc-MBL@Fe3O4 Enrichment and Matrix-Assisted Laser Desorption Ionization–Time of Flight Mass Spectrometry Profiling
Source: Microbiol Spectr. 2022 Nov 8;10(6):e01767-22. doi: 10.1128/spectrum.01767-22 (PMC9769506; doi:10.1128/spectrum.01767-22)
Supplement: Supplemental file 1 — Supplemental material. Download spectrum.01767-22-s0001.pdf, PDF file, 1.2 MB [file spectrum.01767-22-s0001.pdf]

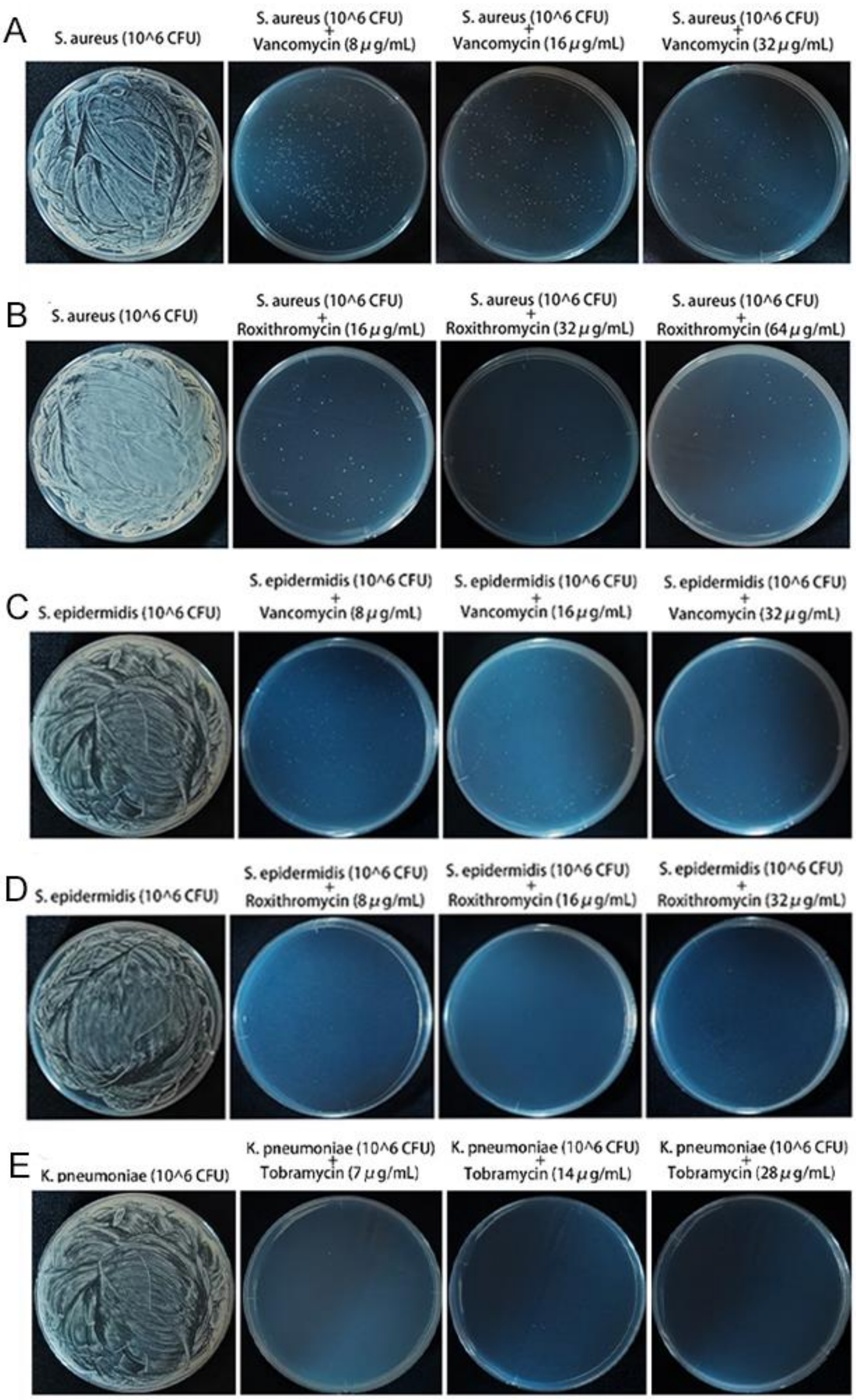

Figure S1. Photographs of culture plates of the original solution and bacterial solution treated with different concentrations of antibiotics. (A) *S. aureus* + vancomycin, (B) *S. aureus* + erythromycin, (C) *S. epidermidis* + vancomycin, (D) *S. epidermidis* + erythromycin, (E) *K. pneumoniae* + tobramycin.

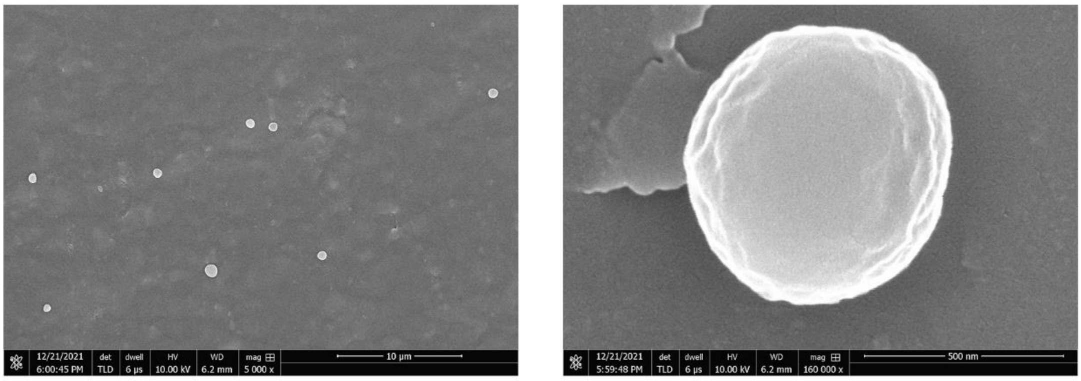

Figure S2. SEM images of *S. aureus* after 24 h of treatment with vancomycin.

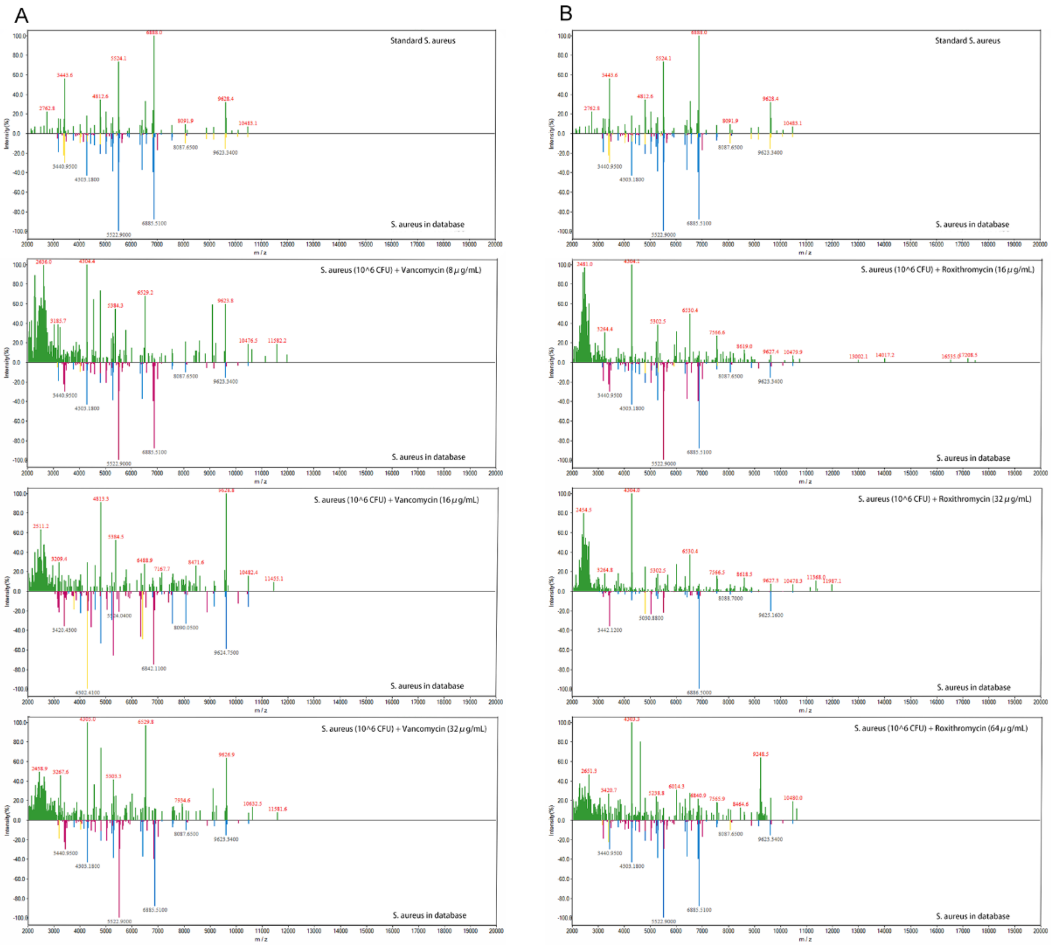

Figure S3. Mass spectra of *S. aureus* obtained from pure solution (top) and enriched by Fc-MBL@Fe3O4 from different concentrations of antibiotic solutions. (A) *S. aureus* + vancomycin, (B) *S. aureus* + erythromycin.

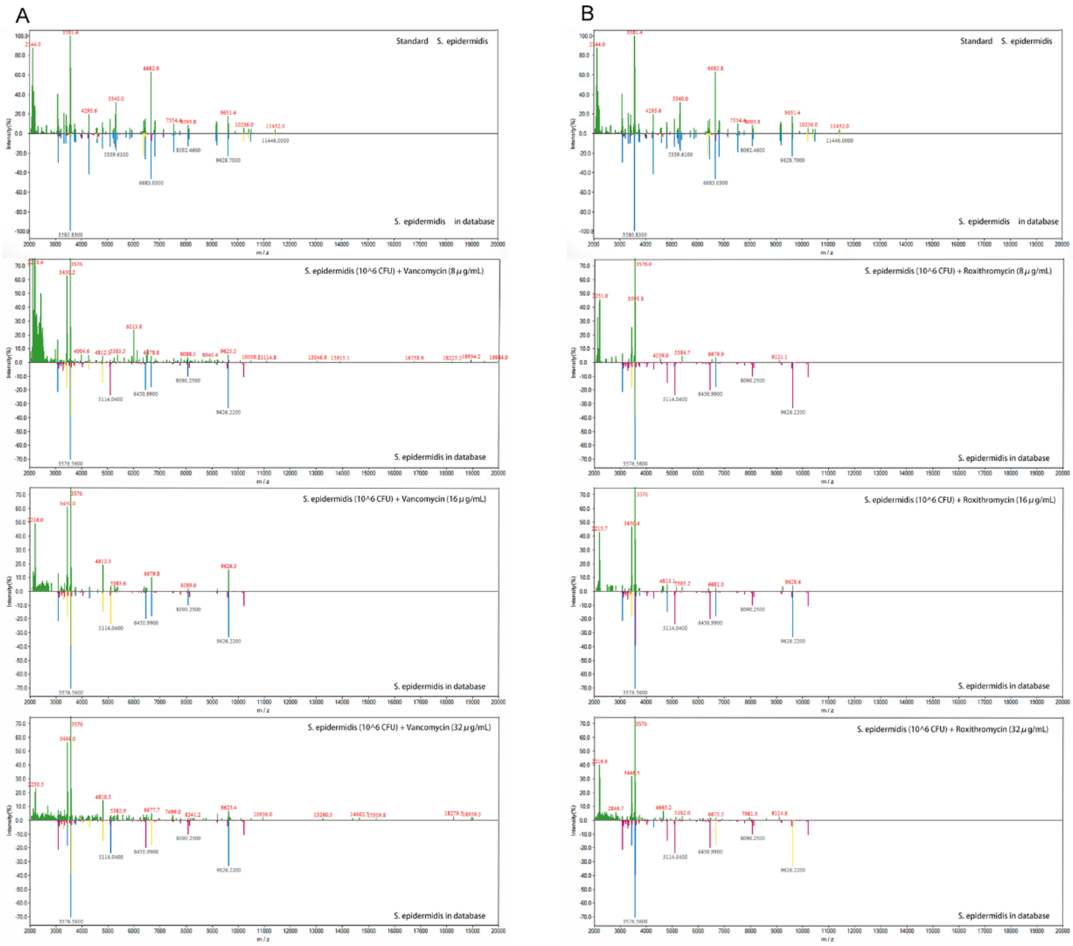

Figure S4. Mass spectra of *S. epidermidis* obtained from pure solution (top) and enriched by Fc-MBL@Fe3O4 from different concentrations of antibiotic solutions. (A) *S. epidermidis* + vancomycin, (B) *S. epidermidis* + erythromycin.

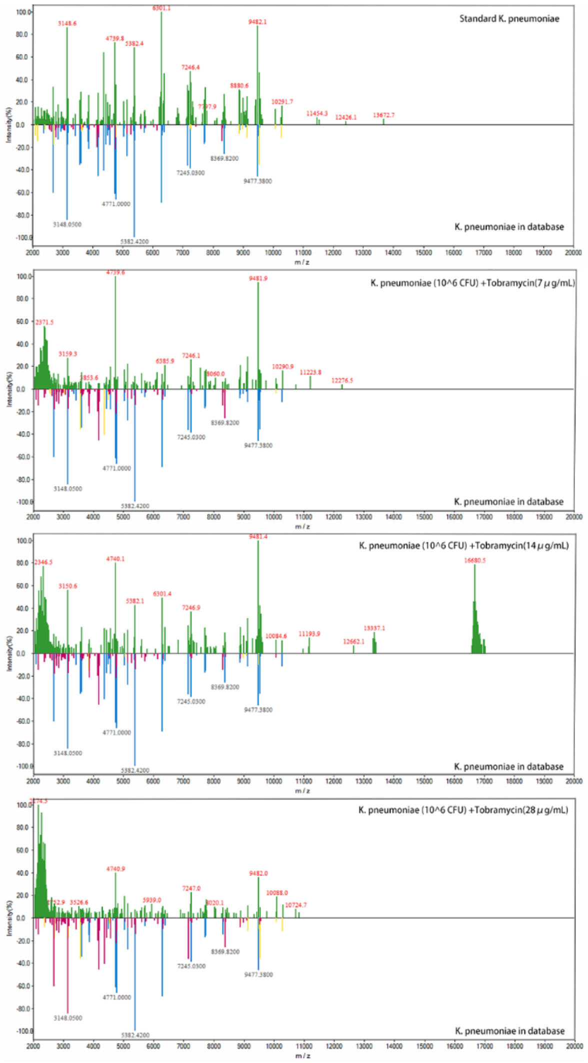

Figure S5. Mass spectra of *K. pneumoniae* obtained from pure solution (top) and enriched by Fc-MBL@Fe3O4 from different concentrations of tobramycin solution.

Table S1. Enrichment efficiency of Fc-MBL@Fe3O4 in aqueous humor

	Dilution	The original bacterial solution (CFU)	Bacteria enriched Fc-MBL@Fe3O4 (CFU)	Supernatant (CFU)	The capture efficiency (%)	Mean ± standard deviation
S. aureus	10^{-3}	8500*	8343*	157	98.2%	99.1±0.8
		8500*	8467*	33	99.6%	
		8500*	8449*	51	99.4%	
	10^{-4}	850*	829*	21	97.5%	97.8±0.4
		850*	830*	20	97.6%	
		850*	835*	15	98.2%	
	10^{-5}	71	119	4	96.7%	98.1±1.6
		78	89	2	97.8%	
		107	149	0	99.9%	
S. epidermidis	10^{-3}	1570*	1564*	6	99.6%	99.5±0.2
		1570*	1559*	11	99.3%	
		1570*	1566*	4	99.7%	
	10^{-4}	134	258	2	99.2%	99.3±0.6
		226	249	3	98.8%	
		112	148	0	99.9%	
	10^{-5}	20	19	1	95%	98.3±2.8
		15	20	0	99.9%	
		29	18	0	99.9%	
K. pneumoniae	10^{-3}	1380*	1352*	28	98.0%	96.4±1.8
		1380*	1336*	44	96.8%	
		1380*	1304*	76	94.5%	
	10^{-4}	165	161	23	87.5%	87.5±5.0
		39	123	10	92.5%	
		209	71	15	82.6%	
	10^{-5}	5	8	0	99.9%	95.2±4.1
		11	14	1	93.3%	
		11	24	2	92.3%	
*: Too many colonies to count. Estimated by diluted sample and dilution ratio						
The capture efficiency (%) = Bacteria enriched Fc-MBL@Fe3O4/ (Bacteria enriched Fc-MBL@Fe3O4 + Supernatant)						

Table S2. Scores of MALDI-TOF MS analysis of different numbers of *S. aureus*.

S. aureus								
Number of bacteria (CFU)	Enrichment with Fc-MBL@Fe3O4				Centrifugation enrichment			
	Five parallel	Group1	Group2	Group3	Five parallel	Group1	Group2	Group3
1.76×10^7	1	2.37	2.36	2.39	1	2.42	2.4	2.32
	2	2.3	2.3	2.44	2	2.38	2.39	2.43
	3	2.38	2.39	2.43	3	2.42	2.45	2.32
	4	2.39	2.44	2.43	4	2.34	2.43	2.3
	5	2.36	2.44	2.35	5	2.47	2.35	2.46
8.80×10^6	1	2.46	2.38	2.35	1	2.47	2.4	2.41
	2	2.4	2.34	2.42	2	2.47	2.41	2.4
	3	2.4	2.45	2.49	3	2.45	2.37	2.45
	4	2.4	2.35	2.41	4	2.36	2.38	2.35
	5	2.36	2.33	2.27	5	2.43	2.43	2.45
4.40×10^6	1	2.48	2.5	2.4	1	1.14	2.2	1.13
	2	2.52	2.51	2.46	2	1.47	1.14	1.8
	3	2.46	2.47	2.49	3	1.53	1.28	1.14
	4	2.48	2.46	2.46	4	1.65	1.82	1.42
	5	2.35	2.41	2.44	5	1.56	1.25	1.33
2.20×10^6	1	2.28	1.94	1.46	1	1.22	1.2	1.4
	2	2.1	2.25	2.24	2	1.34	1.3	1.22
	3	1.5	1.47	2.18	3	1.37	1.23	1.34
	4	1.39	2.21	2.21	4	1.35	1.27	1.36
	5	2.16	1.87	1.81	5	1.34	1.34	1.38
1.10×10^6	1	1.34	1.4	1.29	1	1.45	1.27	1.31
	2	1.4	1.32	1.36	2	1.51	1.25	1.32
	3	1.45	1.33	1.39	3	1.32	1.23	1.51
	4	1.55	1.45	1.47	4	1.42	1.26	1.37
	5	1.62	1.69	1.62	5	1.43	1.26	1.39

Scores of standard *S. aureus*:2.5, 2.6, 2.5, 2.6, 2.6

"Standard *S. aureus*" refers to colonies smeared directly on the target plate for identification by mass spectrometry.

a score >2.0 is considered reliable in the species level, a score 1.7–2.0 indicates identification in the genus level, and a score <1.7 indicates unreliable result.

Table S3. Scores of MALDI-TOF MS analysis of different numbers of *S. epidermidis*.

S. epidermidis								
Number of bacteria (CFU)	Enrichment with Fc-MBL@Fe3O4				Centrifugation enrichment			
	Five parallel	Group1	Group2	Group3	Five parallel	Group1	Group2	Group3
1.19×10^{7}	1	2.06	2.36	2.52	1	2.28	2.11	2.39
	2	2.3	2.36	2.49	2	2.5	2.53	2.49
	3	2.09	2.32	2.39	3	2.27	2.24	2.27
	4	2.28	2.37	2.41	4	2.55	2.51	2.43
	5	2.32	2.59	2.41	5	2.37	2.31	2.41
5.95×10^{6}	1	2.5	2.46	2.43	1	2.43	2.38	1.94
	2	2.37	2.41	2.44	2	2.04	2.11	1.93
	3	2.32	2.43	2.39	3	2.36	2.31	1.85
	4	2.25	2.38	2.39	4	2.49	1.95	1.8
	5	2.16	2.46	2.32	5	2.22	1.98	2
2.98×10^{6}	1	2.42	2.37	2.33	1	1.37	1.17	1.3
	2	2.41	2.39	2.37	2	1.33	1.56	1.34
	3	2.36	2.4	2.38	3	1.34	1.3	1.34
	4	2.39	2.44	2.43	4	1.34	1.43	1.33
	5	2.31	2.41	2.48	5	1.3	1.34	1.37
1.49×10^{6}	1	1.22	2.2	2.26	1	1.36	1.39	1.25
	2	1.53	1.36	1.22	2	1.41	1.27	1.47
	3	1.42	2.13	1.19	3	1.25	1.47	1.12
	4	1.19	2.23	2.18	4	1.45	1.53	1.29
	5	1.78	1.51	1.36	5	1.36	1.36	1.29
7.44×10^{5}	1	1.36	1.4	1.17	1	1.05	1.21	1.27
	2	1.17	1.33	1.23	2	1.4	1.12	1.43
	3	1.19	1.34	1.34	3	1.3	1.33	1.45
	4	1.35	1.14	1.23	4	1.23	1.25	1.37
	5	1.43	1.49	1.2	5	1.37	1.27	1.49

Scores of standard *S. epidermidis*: 2.6, 2.4, 2.5, 2.6, 2.6

"Standard *S. epidermidis*" refers to colonies smeared directly on the target plate for identification by mass spectrometry.

a score >2.0 is considered reliable in the species level, a score 1.7–2.0 indicates identification in the genus level, and a score <1.7 indicates unreliable result.

Table S4. Scores of MALDI-TOF MS analysis of different numbers of *K. pneumoniae*.

K. pneumoniae								
Number of bacteria (CFU)	Enrichment with Fc-MBL@Fe3O4				Centrifugation enrichment			
	Five parallel	Group1	Group2	Group3	Five parallel	Group1	Group2	Group3
1.43×10^{7}	1	2.41	2.46	2.39	1	2.37	2.2	2.22
	2	2.4	2.32	2.4	2	2.39	2.31	2.22
	3	2.42	2.26	2.33	3	2.37	2.27	2.33
	4	2.42	2.44	2.42	4	2.38	2.25	2.31
	5	2.41	2.41	2.37	5	2.34	2.24	2.29
7.15×10^{6}	1	2.28	2.26	1.78	1	2.33	2.34	2.23
	2	2.25	2.14	1.92	2	2.4	2.39	2.3
	3	2.38	2.18	1.89	3	2.32	2.26	2.24
	4	2.35	2.28	2.24	4	2.32	2.19	2.17
	5	2.43	2.11	2.25	5	2.08	2.02	2.03
3.57×10^{6}	1	2.31	2.06	2.29	1	1.8	1.34	1.34
	2	2.24	2.13	2.04	2	1.25	1.46	1.54
	3	2.11	2.08	2.18	3	1.37	1.37	1.43
	4	1.76	1.81	1.9	4	1.54	1.37	1.25
	5	1.79	1.87	1.9	5	1.67	1.47	1.62
1.78×10^{6}	1	2.18	2.13	1.73	1	1.4	1.06	1.4
	2	2.23	2.2	2.24	2	1.24	1.27	1.17
	3	2.35	2.25	1.2	3	1.35	1.19	1.21
	4	2.34	2.27	1.48	4	1.42	1.25	1.24
	5	2.26	2.21	2.27	5	1.45	1.34	1.27
8.94×10^{5}	1	1.34	1.31	1.15	1	1.17	1.14	1.07
	2	1.42	1.49	1.34	2	1.12	1.15	1.14
	3	1.42	1.25	1.42	3	1.15	1.16	1.19
	4	1.36	1.34	1.54	4	1.18	1.21	1.25
	5	1.57	1.38	1.39	5	1.15	1.21	1.21

Scores of standard *K. pneumoniae:* 2.5, 2.4, 2.4, 2.5, 2.5

"Standard *K. pneumoniae*" refers to colonies smeared directly on the target plate for identification by mass spectrometry.

a score >2.0 is considered reliable in the species level, a score 1.7–2.0 indicates identification in the genus level, and a score <1.7 indicates unreliable result.

Table S5. Scores of MALDI-TOF MS analysis of bacteria in aqueous humor after antibiotic action.

	Erythromycin						Vancomycin					
	Concentration	Score					Concentration	Score				
Staphylococcus aureus	16 μg/ml	1.36	2.2	2.1	1.5	2.0	8 μg/ml	1.91	1.79	1.75	1.75	1.75
	32 μg/ml	2.2	1.25	1.31	2.0	2.1	16 μg/ml	1.77	2.0	1.76	1.31	1.71
	64 μg/ml	2.2	1.87	2.1	2.0	1.83	32 μg/ml	2.1	1.86	1.8	1.97	1.76
Staphylococcus epidermidis	8 μg/ml	1.75	1.68	1.73	1.8	2.0	8 μg/ml	2.1	1.56	1.98	1.87	1.33
	16 μg/ml	1.9	1.3	1.42	1.65	1.85	16 μg/ml	2.2	2.3	2.4	2.3	2.3
	32 μg/ml	1.7	1.48	1.71	1.7	1.7	32 μg/ml	1.96	2.2	2.2	2.1	1.94
	Tobramycin											
	Concentration	Score										
Klebsiella pneumoniae	7 μg/ml	2.3	2.5	2.4	2.2	2.4						
	14 μg/ml	2.4	2.2	2.3	2.5	2.3						
	28 μg/ml	2.4	2.3	2.2	2.2	2.3						

a score >2.0 is considered reliable in the species level, a score 1.7–2.0 indicates identification in the genus level, and a score <1.7 indicates unreliable result.

Table S6. Scores of MALDI-TOF MS analysis of micro-LB broth for short-term incubation of bacteria.

	Pre-incubation (CFU)	5h					6h				
		Score					Score				
S. aureus + 10 µl LB	49	1.22	1.28	1.31	1.33	1.45	2.1	2.4	2.4	2.2	2.2
	80	2.3	1.53	2.0	1.36	1.7	2.4	2.4	2.5	2.4	2.5
	52	1.67	2.02	1.94	1.34	2.3	2.11	2.29	2.32	2.3	2.34
S. aureus + 20 µl LB	49	2.3	2.4	1.78	2.3	2.3	2.3	2.4	2.3	2.3	2.4
	80	2.4	2.3	2.4	2.3	2.4	2.4	2.4	2.4	2.4	2.3
	52	2.24	1.8	2.28	2.27	2.34	2.27	2.08	2.06	2.01	1.80
S. aureus + 30 µl LB	49	1.31	1.05	1.22	1.39	1.53	2.4	2.4	2.5	2.5	2.4
	80	2.3	2.4	2.5	2.4	2.4	2.3	2.2	2.5	2.3	2.4
	52	2.23	2.25	1.20	1.23	1.23	2.34	2.19	1.88	1.99	2.19
K. pneumoniae + 10 µl LB	21	1.17	1.19	1.22	1.22	1.16	2.4	1.19	1.86	2.1	1.71
	34	1.17	1.72	1.50	2.2	2	2.5	2.5	2.5	2.5	2.5
	39	1.19	1.69	1.36	1.39	1.58	2.37	2.42	2.43	2.43	2.54
K. pneumoniae + 20 µl LB	21	1.83	1.5	1.82	1.14	1.65	2.1	2.1	2.2	2.3	2.1
	34	1.3	1.22	1.25	1.28	1.42	2.5	2.5	2.4	2.5	2.4
	39	1.34	2.25	1.36	1.47	1.48	2.41	2.41	2.42	2.46	2.48
K. pneumoniae + 30 µl LB	21	1.86	1.17	1.22	1.31	1.34	2.3	2.3	2.3	2.2	2.5
	34	1.36	1.38	1.39	1.39	1.25	2.1	2.1	2.3	2.3	2.4
	39	1.12	1.22	1.25	1.31	1.33	1.76	2.34	2.35	2.43	2.53
	Pre-incubation (CFU)	7h					8h				
		Score					Score				
S. epidermidis + 10 µl LB	171	1.19	1.14	1.14	1.14	1.39	2	2.1	1.48	1.66	1.6
	216	1.6	1.25	1.31	1.36	1.39	2	1.56	2	1.79	1.67
	282	1.42	1.42	1.1	n/a	1.67	1.85	2.0	1.39	1.9	1.65
S. epidermidis + 20 µl LB	171	1.84	1.98	1.25	1.7	1.33	2	1.67	2.1	2.1	2.2
	216	1.2	1.19	1.26	1.26	1.34	2.1	2.1	2.2	2.1	2.2
	282	1.28	1.72	1.48	1.62	n/a	2.17	2.17	2.18	2.23	2.25
S. epidermidis + 30 µl LB	171	1.17	1.17	1.19	1.28	1.53	2.2	2.2	1.85	2.1	1.98
	216	2.1	1.6	1.34	2	1.62	2.2	2.3	2.1	2.2	2.2
	282	1.7	1.17	n/a	1.36	1.45	1.79	2.16	2.26	1.88	2.28

a score >2.0 is considered reliable in the species level, a score 1.7–2.0 indicates identification in the genus level, and a score <1.7 indicates unreliable result.; n/a = not applicable.

Table S7. OD values for bacterial solutions containing different volumes of LB broth for 4 – 10 h.

OD value													
		S. aureus			*S. epidermidis*			*K. pneumoniae*			Control group		
Time		10 μl LB	20 μl LB	30 μl LB	10 μl LB	20 μl LB	30 μl LB	10 μl LB	20 μl LB	30 μl LB	10 μl LB	20 μl LB	30 μl LB
4h	Mean	0.023	0.027	0.017	0.000	0.007	0.010	0.010	0.017	0.020	0.023	0.037	0.047
	SD	0.006	0.006	0.006	0.000	0.012	0.000	0.000	0.012	0.000	0.006	0.006	0.012
	P	0.179			0.135			0.135			0.095		
5h	Mean	0.233	0.193	0.117	0.010	0.040	0.027	0.177	0.223	0.193	0.027	0.040	0.020
	SD	0.023	0.023	0.023	0.010	0.017	0.025	0.015	0.040	0.035	0.006	0.010	0.010
	P	0.028[b]			0.162			0.141			0.099		
6h	Mean	1.237	0.903	0.643	0.010	0.037	0.050	0.770	0.917	0.847	0.020	0.020	0.020
	SD	0.049	0.065	0.042	0.000	0.023	0.026	0.030	0.025	0.076	0.010	0.010	0.000
	P	0.022[b]			0.076			0.079			0.859		
7h	Mean	2.373	2.217	2.143	0.043	0.150	0.053	0.913	1.303	1.333	0.023	0.020	0.037
	SD	0.078	0.081	0.025	0.040	0.026	0.021	0.107	0.179	0.042	0.006	0.000	0.006
	P	0.036[b]			0.059			0.061			0.087		
8h	Mean	3.210	3.383	3.550	0.543	0.737	0.500	1.737	2.380	2.327	0.033	0.047	0.027
	SD	0.026	0.164	0.070	0.064	0.029	0.078	0.101	0.182	0.021	0.015	0.006	0.006
	P	0.051			0.054			0.061			0.141		
9h	Mean	3.887	4.060	4.270	1.183	2.180	1.270	2.377	2.860	2.850	0.020	0.033	0.043
	SD	0.206	0.101	0.122	0.012	0.170	0.087	0.101	0.114	0.061	0.010	0.012	0.012
	P	0.079			0.054			0.063			0.122		
10h	Mean	4.327	5.060	5.073	2.527	3.613	3.680	2.447	3.283	3.380	0.053	0.053	0.053
	SD	0.216	0.066	0.055	0.185	0.103	0.208	0.042	0.112	0.095	0.006	0.012	0.012
	P	0.063			0.061			0.034[b]			0.96		

Based on data type and distribution, Kruskal-Wallis test was used to compare different volumes of LB broth.

a: (bacteria + 10μl aqueous humour + 10μl LB) VS (bacteria + 10μl aqueous humour + 20μl LB)

b: (bacteria + 10μl aqueous humour + 10μl LB) VS (bacteria + 10μl aqueous humour + 30μl LB)

c: (bacteria + 10μl aqueous humour + 20μl LB) VS (bacteria + 20μl aqueous humour + 30μl LB)
